# Genomic characterization of *Pseudomonas aeruginosa* adaptation mechanisms in bronchiectasis patients

**DOI:** 10.1515/almed-2025-0044

**Published:** 2025-08-15

**Authors:** Alba Muñoz Santa, Xavier Gómez-Arbonés, Ricardo Pifarré Teixidó, Mercè García-González, Alba Bellés Bellés

**Affiliations:** Servicio de Análisis Clínicos, Hospital Universitari Arnau de Vilanova de Lleida, Lleida, Spain; Institut de Recerca Biomèdica de Lleida (IRB Lleida), Universitat de Lleida, Lleida, Spain; Departamento de Medicina y Cirugía, Institut de Recerca Biomèdica de Lleida (IRB Lleida), Universitat de Lleida, Lleida, Spain; Servicio de Neumología, Hospital Universitari Arnau de Vilanova de Lleida, Lleida, Spain

**Keywords:** bronchiectasis, *Pseudomonas aeruginosa*, genomic adaptation

## Abstract

**Objectives:**

*Pseudomonas aeruginosa* is one of the main causes of chronic bronchial infection (CBI), especially in patients with chronic underlying diseases such as cystic fibrosis (CF), chronic obstructive pulmonary disease (COPD), asthma and bronchiectasis (BQ). Compared to *P. aeruginosa* CBI in CF, BQ infection has historically received less attention. The aim of this study was to determine the antibiotic susceptibility profile of 100 isolates recovered from 100 patients with *P. aeruginosa* CBI BQ and to characterize some of the adaptation mechanisms in 55 isolates by whole genome sequencing (WGS).

**Methods:**

Susceptibility testing to 10 antipseudomonal agents was done by MicroScan WalkAway broth microdilution system. WGS was performed using the Illumina DNA Prep library preparation kit. Indexed libraries were sequenced on an Illumina MiSeq benchtop sequencer (300 base pairs paired-end reads).

**Results:**

The most common loss-of-function mutations occurred in genes encoding the MexAB-OprM efflux-pump system, the pvd cluster and the fpvA receptor, and genes involved in twitching motility such as *chpA* and *fimV*.

**Conclusions:**

Our data indicates that *P. aeruginosa* adapts by accumulating loss-of-function mutations in several genes, resulting in changes to different phenotypes that may guide the development of new alternative treatment therapies.

## Introduction

*Pseudomonas aeruginosa* is an opportunistic pathogen responsible for hospital-acquired infections, particularly in immunocompromised patients. Additionally, *P. aeruginosa* is a leading cause of chronic bronchial infections (CBI), contributing to morbidity and mortality in chronic inflammatory diseases, such as cystic fibrosis (CF), chronic obstructive pulmonary disease (COPD) or bronchiectasis (BQ) [1].

BQ is characterized by the permanent dilation and progressive destruction of the bronchial walls, caused by a variety of systemic and local diseases [2].

Limited data has been published regarding genomic characterization of *P. aeruginosa* in patients with BQ [3]. Most genomic studies to date have focused on antibiotic resistance mechanisms [4].

The aim of this study was to determine the antibiotic susceptibility of *P. aeruginosa* isolates recovered from patients with non-CF BQ and to characterize other genetic adaptive mechanisms beyond antibiotic resistance that contribute to the persistence of this pathogen in CBI.

## Materials and methods

### *P. aeruginosa* collection and antibiotic susceptibility testing

The studied collection comprised 100 *P. aeruginosa* isolates recovered from respiratory samples of 100 different patients with non-CF BQ who attended consecutively the Hospital Universitari Arnau de Vilanova de Lleida (Catalonia, Spain) between September 2019 and May 2021.

Patients included were diagnosed with bronchiectasis based on high-resolution computed tomography (HRCT) findings and compatible clinical symptoms. The most frequent etiologies identified in this cohort were COPD (52 %), idiopathic causes (26 %), other pulmonary conditions (18 %), and genetic diseases such as primary ciliary dyskinesia (PCD) (3 %) and Kartagener syndrome (1 %).

MICs of aztreonam, piperacillin/tazobactam, ceftazidime, cefepime, imipenem, meropenem, tobramycin, amikacin, ciprofloxacin, and colistin were determined by broth microdilution using the MicroScan WalkAway^®^ system (Siemens, Healthcare). E-test gradient strips (bioMérieux) were used to determine the MIC of *P. aeruginosa* isolates with mucoid phenotype and/or showing hypersusceptibility antibiotic profile. EUCAST v14.0 clinical breakpoints were applied for interpretation of S/I/R categories and ECDC-established recommendations were used to define MDR profiles [5].

### Genomic characterization of *P. aeruginosa* isolates

A total of 55 isolates were selected for sequencing based on phenotypic diversity (mucoid, non-mucoid, and small colony variant (SCV) and differing antibiotic susceptibility profiles, given that most *P. aeruginosa* isolates in bronchiectasis are generally susceptible. Isolates producing carbapenemases or with brown pigmentation were excluded, as they likely represent already adapted subpopulations. This approach aimed to represent the genetic variability of *P. aeruginosa* associated with bronchiectasis.

Total genomic DNA was extracted using a commercially available kit (QIAsymphony DSP DNA Kit, QIAGEN) through automated extraction on the QIAsymphony system (QIAGEN). Indexed paired-end libraries were prepared (Illumina DNA Prep, Illumina) and then sequenced on an Illumina MiSeq^®^ using the MiSeq reagent kit v3 and 600 cycles. For the genomic characterization, 300 bp paired-ended reads were *de novo* assembled with SPAdes using default options in order to infer the sequence type (ST) (MLST v2.0.4., available at http://www.genomicepidemiology.org/services). A variant calling analysis was performed using the Snippy v.3.1 [6] with the PAO1 genome (NC_002516.2) as the reference. Default parameters were applied, including a minimum mapping quality of 60, a base quality threshold of 13, a minimum read coverage of 10, and a requirement that at least 90 % of the reads support a variant for it to be called. Large deletions in *P. aeruginosa* genome were detected with the Integrative Genomics Viewer (IGV) tool.

## Results

**Figure 1:**
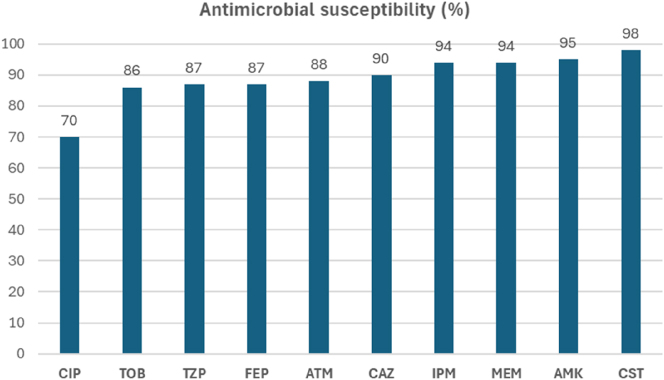
Antimicrobial susceptibility (%) of the *P. aeruginosa* BQ collection. CIP, ciprofloxacin; TOB, tobramycin; TZP, piperacillin/tazobactam; FEP, cefepime; ATM, aztreonam; CAZ: ceftazidime; IPM, imipenem; MEM, meropenem; AMK, amikacin; CST, colistin.

Figure 1 shows the antimicrobial susceptibility percentages of the 100 *P. aeruginosa* isolates.

Higher resistance rates were documented for ciprofloxacin, whereas colistin, amikacin and carbapenems were the most active compounds. Most of the sequenced isolates were classified as hypersusceptible to nearly all tested antibiotics, except for seven isolates identified as MDR. These MDR isolates are indicated in Table 1, which shows the antibiotic susceptibility profile of all sequenced isolates.

**Table 1:** Antibiotic susceptibility profile of all 55 sequenced isolates.

MIC (mg/L)^a^											
Isolate ID	ST	ATM	TZP*	CAZ	FEP	IMI	MER	TOB	AK^b^	CIP	CST
BQ01	1251	0.75	≤8	1.5	4	0.75	0.125	2	≤8	1.5	1
BQ02	244	0.016	≤8	0.047	4	0.75	0.5	0.032	≤8	>32	1
BQ03	155	0.125	≤8	0.38	3	0.50	3	0.75	≤8	0.016	1.5
BQ04	242	1.5	≤8	1.5	4	2	0.38	2	16	1.5	1
BQ05	262	3	≤8	1.5	4	0.75	0.25	1	≤8	1.5	1
BQ06	395	3	≤8	3	0,75	6	2	0.28	≤8	0.38	3
BQ07^c^	348	12	≤8	24	0,75	1.5	0.75	8	32	1	1
BQ08	598	0.25	≤8	1	1,5	0.75	0.38	0.75	≤8	0.38	1
BQ09	170	0.19	≤8	0.5	0,75	1.5	0.125	1.5	≤8	0.064	2
BQ10	3,016	1.5	≤8	1	4	0.75	0.25	1	≤8	0.5	1.5
BQ11^c^	UK	0.50	24	2	16	0.75	0.064	2	≤8	3	1
BQ12	395	0.25	≤8	1	8	1	0.064	1.5	16	0.38	1
BQ13	253	0.25	≤8	0.50	≤1	1.5	0.125	1.5	≤8	>32	1
BQ14	633	1.5	≤8	1	4	2	0.50	2	≤8	2	2
BQ15	2,431	2	64	1	1	2	0.125	1	≤8	0.094	2
BQ16^c^	175	12	64	16	12	>32	>32	>256	≤8	>32	2
BQ17	532	3	≤8	0.75	1	1.50	1	0.75	≤8	1	1
BQ18	554	0.25	≤8	0.50	4	4	1	0.75	≤8	0.50	2
BQ19	253	1	≤8	0.50	0.75	1	0.38	1	≤8	0.064	1
BQ20	UK	0.094	≤8	0.125	0.047	0.064	0.008	0.50	≤8	0.008	1
BQ21	480	0.38	≤8	0.38	0.5	0.38	0.064	0.064	≤8	0.19	1.5
BQ22	UK	0.38	≤8	1	1.5	3	0.032	1.5	≤8	0.064	1
BQ23	357	4	≤8	1	2	1	0.125	2	≤8	0.125	3
BQ24	2,431	0.5	≤8	0.5	1	0.75	0.064	1.5	≤8	0.094	1.5
BQ25	1,600	2	≤8	1	1	3	0.75	1	≤8	0.125	1
BQ26^c^	UK	1.5	64	6	2	>32	>32	0.50	≤8	>32	1
BQ27	379	2	≤8	0.75	0.75	1.5	0.19	1	≤8	0.094	2
BQ28^c^	633	>256	≤8	8	>256	1.5	0.75	>256	≤8	>32	2
BQ29	274	0.047	≤8	0.094	0.25	0.25	0.012	0.047	≤8	2	0.25
BQ30	253	0.25	≤8	0.38	0.75	0.38	0.047	0.75	≤8	0.023	1
BQ31	1,637	3	≤8	1	1	1	0.25	1	≤8	0.094	1.5
BQ32	253	4	≤8	1	1	1	0.50	1	≤8	0.094	4
BQ33	360	0.50	≤8	2	12	1	0.049	3	32	0.25	1
BQ34	1,002	0.38	≤8	0.75	0.20	1	0.094	0.75	≤8	0.035	2
BQ35	3,119	3	≤8	1.5	16	0.19	0.094	2	32	0.25	1
BQ36	262	4	≤8	3	3	3	0.50	2	≤8	0.125	3
BQ37	447	2	≤8	1	1.5	2	0.50	1	≤8	0.094	1.5
BQ38	UK	3	≤8	1	1	1.5	0.125	1	≤8	0.023	1
BQ39	585	0.19	≤8	0.75	0.25	1.5	0.023	0.25	≤8	0.023	1
BQ40	3,218	1.5	≤8	0.75	1	0.75	0.094	0.75	≤8	0.064	1
BQ41	598	0.19	≤8	0.5	6	0.38	0.19	0.25	≤8	0.38	1
BQ42	395	0.094	≤8	0.125	0.094	0.094	0.012	0.064	≤8	0.008	1
BQ43	17	0.25	≤8	0.094	0.125	0.125	0.012	0.094	≤8	0.047	0.064
BQ44	UK	2	≤8	0.75	0.75	0.75	0.064	0.125	≤8	0.047	0.064
BQ45	253	0.380	≤8	0.75	8	0.250	0.064	3	32	0.5	2
BQ46	792	0.75	≤8	0.38	2	1	0.094	0.75	≤8	0.38	1
BQ47	3,511	16	≤8	3	4	2	8	0.125	≤8	2	2
BQ48	1,393	4	≤8	2	4	2	0.38	1	≤8	0.125	1.5
BQ49	455	0.38	≤8	1	0.25	2	0.094	1	≤8	0.064	1
BQ50	253	2	≤8	1	8	1.5	0.5	1	≤8	>32	1.5
BQ51	253	4	≤8	1	0.094	1	0.75	1.5	≤8	>32	1
BQ52	27	0.125	≤8	0.25	4	0.125	0.094	0.5	32	>32	1
BQ53^c^	UK	64	>256	96	24	2	0.75	1	≤8	0.064	1
BQ54	274	3	≤8	4	4	1.5	0.75	1	≤8	0.50	1
BQ55^c^	235	48	64	32	24	1	0.75	2	≤8	>32	1

Minimum inhibitor concentrations (MIC) (mg/L)^a^ of ATM, aztreonam (S≤0.001; R>16); TZP, piperacillin/tazobactam (S≤0.001; R>16); CAZ, ceftazidime (S≤0.001; R>8); FEP, cefepime (S≤0.001; R>8); IMI, imipenem (S≤0.001; R>4); MER, meropenem (S≤2; R>8); TOB, tobramycin (S≤2; R>2); AK, amikacin (S≤16; R>16); CIP, ciprofloxacin (S≤0.001; R>0.5); CST, colistin (S≤4; R>4). MICs were determined by E-test, except those marked with ^b^, which were determined by broth microdilution. Green colour indicates clinical susceptibility at standard and increased dosing, while red indicates resistance. ^c^Indicates multidrug-resistant (MDR) isolates. ST column represents the sequence type. “UK” in the ST column means unkown.

In terms of characterizing other adaptability mechanisms, variant calling analysis was performed on 55 sequenced isolates to identify loss-of-function mutations.

Table 2 shows STs and the null mutations identified in different genes associated with *P. aeruginosa* adaptability to the chronic pulmonary environment.

**Table 2:** Sequence type (ST) and loss-of-function mutations identified in MexAB-OprM efflux-pump system, *mutS*-*mutL, mucA-mucB;* psl cluster*, bifA-rbdA-oprF-ladS* and *pilJ-chpA-fimV* genes, for each isolate.

Isolate ID	ST	MexAB-OprM	*mutS-mutL*	*mucA-mucB*	psl cluster	*bifA-rbdA-oprF-ladS*	*pilJ-chpA-fimV*
BQ01^b^	1251	*mexA* (nt30Δ2)	–	*mucA* (Q123X)	–	–	*chpA* ^c^ *, fimV* ^c^
BQ02^b^	244	*oprM* (Q246X)	–	*mucA* (Q118X)	ΔpslA-pslO (≈104 kb)	–	–
BQ03^b^	155	–	–	*mucA* (V147X)	ΔpslA-pslO (≈135 kb)	–	–
BQ04^a^	242	–	–	*mucA* (A90X)	–	–	–
BQ05^a^	262	–	–	*mucA* (Q117X)	ΔpslE-pslN (≈132 kb)	*oprF* (nt576Δ5, nt844Δ1)	–
BQ06^a,b^	395	*oprM* (nt800Δ1)	–	*mucA* (H159X)	ΔpslA-pslO (≈142 kb)	–	–
BQ07^a^	348	*oprM* (aas202InsHis)	–	*mucA* (V99X)	ΔpslB-pslC (≈1.8 kb)	*ladS* (aas497Δ6)	–
BQ08^a^	598	*mexA* (Q107X)	–	*mucA* (V147X)	–	–	–
BQ09	170	*mexA* (Q295X)	–	–	–	–	–
BQ10	3,016	–	*mutL* (aas383Δ1)	–	–	–	*chpA* ^c^ *, fimV* ^c^
BQ11	UK	*mexA* (Q183X)	*mutL* (nt819Δ1)	–	–	–	*pilJ* (nt1122Δ2), *chpA*^c^*, fimV*^c^
BQ12	395	*mexB* (nt2621Δ1)	–	–	–	–	–
BQ13	253	*mexA* (Q46X)	–	–	ΔpslA-pslO (≈278 kb)	*rbdA* (nt190Δ1), *oprF* (Q160X)	*chpA* ^c^ *, fimV* ^c^
BQ14	633	*mexB* (nt41InsGATC)	–	–	–	–	–
BQ15	2,431	–	–	–	–	–	–
BQ16	175	–	–	–	–	–	–
BQ17	532	*oprM* (nt1274Δ1)	–	–	–	*rbdA* (Y366X)	*chpA* ^c^
BQ18	554	–	–	–	–	–	*fimV* ^c^
BQ19	253	–	–	–	–	*rbdA* (nt190Δ1), *ladS* (W4X)	*chpA* ^c^ *, fimV* ^c^
BQ20	UK	*mexB* (nt32Δ1)	–	*mucA* (aas56Δ138)	ΔpslA-pslO (≈163 kb)	*rbdA* (aas208Δ1), *oprF* (Q27X)	–
BQ21^b^	480	*oprM* (*nt505*Δ5)	*mutS* (nt1592Δ4)	*mucA* (T96X)	–	–	*fimV* ^c^
BQ22	UK	*mexA* (nt749Δ195)	–	–	–	–	*chpA* ^c^ *, fimV* ^c^
BQ23	357	–	–	–	–	–	*chpA* ^c^
BQ24	2,431	*mexA* (nt19Δ1)	–	–	–	–	*chpA* ^c^ *, fimV* ^c^
BQ25	1,600	–	–	–	–	–	–
BQ26^b^	UK	*mexB* (Q112X)	–	–	–	–	–
BQ27	379	–	*mutS* (nt339Δ1)	–	–	–	*fimV* ^c^
BQ28	633	*mexB* (nt46InsATCG)	–	–	–	–	–
BQ29	274	*mexA* (nt19Δ2)	–	*mucA* (V147X)	–	–	*chpA* ^c^
BQ30	253	*mexB* (nt283InsG)	–	–	ΔpslA-pslO (≈67 kb)	*rbdA* (nt190Δ1)	*chpA* ^c^ *, fimV* ^c^
BQ31	1,637	–	–	–	–	–	*fimV* ^c^
BQ32	253	–	–	*mucB* (L225X)	–	*rbdA* (nt190Δ1)	*chpA* ^c^ *, fimV* ^c^
BQ33	360	*mexB* (nt2169Δ1)	–	–	–	–	*pilJ* (aas672Δ4)
BQ34	1,002	*mexB* (Q439X)	–	–	ΔpslA-pslN (≈28.8 kb)		*chpA* ^c^ *, fimV* ^c^
BQ35	3,119	–	–	–	–	*rbdA* (nt97InsG)	*chpA* ^c^
BQ36	262	–	–	–	–	–	*chpA* ^c^
BQ37	447	–	–	–	–	–	–
BQ38	UK	–	–	–	–	–	*chpA* ^c^ *, fimV* ^c^
BQ39	585	*oprM* (nt505Δ5)	–	–	–	–	*fimV* ^c^
BQ40	3,218	–	–	–	–	*ladS* (nt1345InsAT)	*chpA* ^c^
BQ41^a^	598	*mexA* (Q107X)	–	*mucA* (V147X)	–	–	–
BQ42^a^	395	*oprM* (nt318InsC)	–	*mucA* (V147X)	–	*rbdA* (nt617InsT)	–
BQ43	17	*mexB* (aas739Δ9)	–		–	*ladS* (nt6151Δ1)	*fimV* ^c^
BQ44^a^	UK	–	–	*mucA* (aas191Δ4)	–		*chpA* ^c^ *, fimV* ^c^
BQ45	253	–	–	*mucA* (nt187Δ1)	ΔpslG-pslO (≈9.8 kb)	*rbdA* (nt190Δ1), *ladS* (nt2107Δ1)	*chpA* ^c^ *, fimV* ^c^
BQ46^a^	792	–	–	*mucA* (V129X)	–	–	*fimV* ^c^
BQ47	3,511	–	–	–	–	–	–
BQ48	1,393	–	–	–	–	–	*chpA* ^c^
BQ49	455	*mexB* (Q106X)	–	–	–	–	–
BQ50	253	–	–	–	–	*rbdA* (nt190Δ1), *ladS* (nt1788Δ1)	*chpA* ^c^ *, fimV* ^c^
BQ51	253	–	–	–	–	*rbdA* (nt190Δ1), *ladS* (nt1788Δ1)	*chpA* ^c^ *, fimV* ^c^
BQ52^a^	27	*mexB* (aas766Δ3)	–	*mucA* (V147X)	–	*rbdA* (nt250InsC)	–
BQ53^b^	UK	–	*mutS* (nt2550Δ2)	–	–	–	–
BQ54	274	–	–	–	–	–	*chpA* ^c^
BQ55	235	–	–	–	–	–	*chpA* ^c^ *, fimV* ^c^

^a^Mucoid phenotype, ^b^SCV phenotype, –indicates absence of mutation, and ^c^multiple null mutations. UK in the ST column means unknown.

Among the 55 sequenced isolates, the most frequent ST was ST253 (7), followed by ST395 (3). Seven isolates exhibited a novel allele combination and could not be assigned to any previously described ST. The most common high-risk STs identified were ST175 (1), ST235 (1), ST244 (1) and ST357 (1), with the latter three exhibiting a non-MDR profile. Additionally, other high-risk clones with non-MDR profiles included ST17, ST274, and ST532, each represented by a single isolate.

One of the most frequent loss-of-function mutations was found in the genes encoding the three components of the MexAB-OprM efflux-pump, with at least one of these genes being mutated in 27 isolates.

Hypermutability due to loss-of-function mutations in the DNA mismatch repair genes *mutS* or *mutL*, is a common trait in chronic *P. aeruginosa* isolates. In this non-CF *P. aeruginosa* setting, five isolates presented truncating mutations in these genes.

Regarding the mucoid phenotype, 17 isolates carried truncating mutations in *mucA,* and only one in *mucB*. Of these, 10 exhibited a mucoid phenotype (BQ04-05-06-07-08-41-42-44-46-52). These genes encode proteins that act as negative regulators of the sigma factor AlgU, which is crucial for alginate overproduction and biofilm formation.

The IGV tool was used to detect large deletions in *P. aeruginosa* genome. Large deletions were analysed in the pel, alg and pel gene clusters which are involved in biofilm formation, as well as in genes related to pyoverdine synthesis (pvdABC operon) and its receptor fpvA, both implicated in iron acquisition.

In 10 of these isolates, complete deletion of two or more genes within the psl cluster were detected, ranging from approximately 1.8–278 kb, while the alg and pel gene clusters remained intact in all cases.

Regarding biofilm formation, *bifA*, *rbdA*, *oprF* and *ladS* genes were also analyzed by variant calling analysis.

No mutations were detected in *bifA,* which encodes a c-di-GMP phosphodiesterase that promotes swarming motility over biofilm formation. Conversely, RbdA, another phosphodiesterase involved in c-di-GMP degradation, showed null mutations in 12 isolates*.* Similarly, OprF, the major outer membrane porin in *P. aeruginosa* showed null mutations in three isolates. LadS positively regulates the expression of genes involved in the production of biofilm-associated exopolysaccharides, including Pel and alginate, as well as a broad range of virulence factors, which include genes involved in motility and the expression of the type III secretion system (T3SS). In this study, seven isolates were found to have null mutations in *ladS*.

Regarding the characterization of pyoverdine synthesis and its receptor fpvA, deletion of one or more pvd cluster genes and/or the fpvA receptor gene was detected in all isolates, except for BQ02-06-07-12-22-25-30-32-42-43-44-45-48-50-51-52 (not shown in Table 2).

Other loss-of-function mutations in genes such as *pilJ, chpA* and *fimV* are associated with altered twitching motility, which may be an adaptation related to the viscosity of the sputum environment. In this study, null mutations were detected in two isolates for *pilJ*, 24 isolates for *chpA*, and 23 isolates for *fimV.* Mutations in *chpA* and *fimV* are not shown in Table 2, as there are multiple null mutations in these genes.

## Discussion

These findings suggest that, unlike in other chronic respiratory infections, antibiotic resistance rates are not markedly high in this subset of *P. aeruginosa* causing non-CF BQ. This scenario provides a unique opportunity to explore alternative genetic adaptive mechanisms beyond antibiotic resistance that contribute to the persistence of this pathogen in CBI.

The ST distribution broadly reflects the global *P. aeruginosa* population. The identification of six isolates with novel allele combinations suggests ongoing genetic evolution within this population. Furthermore, the detection of high-risk clones, such as ST175, ST235, ST244, and ST357, mostly linked to non-MDR profiles, highlights the intricate dynamics of clonal dissemination and their potential impact on infection management and therapeutic strategies [7].

Regarding the adaptability mechanisms of *P. aeruginosa* to the chronic pulmonary environment, the genome analysis of 55 *P. aeruginosa* isolates from patients with BQ has revealed significant genetic adaptations that promote the persistence of the bacteria in this patient group.

The highest number of loss-of-function mutations was found on the MexAB-OprM efflux pump, which plays a key role in the basal resistance to most β-lactams, including the novel beta-lactam/beta-lactamase inhibitors combinations. This finding explains the susceptibility profile of these isolates to these antibiotics. Moreover, MexAB-OprM has been implicated in virulence, suggesting that null mutations may be driven by other mechanisms beyond antibiotic efflux [8].

Regarding biofilm formation, the deletion of genes in the psl cluster along with the integrity of the alg and pel clusters, suggests that in chronic *P. aeruginosa* infections in BQ, biofilm formation is primarily driven by the polysaccharides Pel and alginate, rather than Psl [9]. Furthermore, the high prevalence of truncating mutations in the *mucA* gene reinforces the importance of alginate overproduction in the development of the mucoid phenotype, a key trait in the virulence and resistance of the bacteria during chronic infections [10]. The impairment of RbdA and OprF has also been shown to increase c-di-GMP levels, leading to increased biofilm formation through the overproduction of Pel [9], 11].

Mutations in LadS could enhance motility and T3SS activity over Pel and alginate biofilm production [12]. Notably, one of the four isolates carrying a null mutation in this gene also exhibited a complete deletion of *pslB* and *pslC* genes, suggesting that biofilm formation could be significantly impaired in this isolate.

Additionally, the frequent deletion of genes associated with pyoverdine synthesis and its receptor FpvA indicates that *P. aeruginosa* adapts to the host by favoring alternative iron acquisition routes, such as through the heme group of host proteins [13].

Finally, a high number of loss-of-function mutations were observed in genes associated with twitching motility, such as *pilJ, chpA* and *fimV.* These mutations are linked to impaired or amended twitching motility, a phenotype also observed in CF, suggesting that this may be an adaptation to the viscosity of the sputum environment [3], 13].

These genomic insights are crucial for improving therapeutic strategies in bronchiectasis.

For instance, the deletion of the mexAB efflux pump can enhance susceptibility to β-lactam antibiotics, enabling more targeted treatment options [8]; while the loss of pyoverdine biosynthesis suggests a shift to heme uptake, highlighting the potential of iron chelation therapies to disrupt iron metabolism and biofilm formation.

Moreover, given* P. aeruginosa*’s rapid evolution of resistance, which limits the long-term effectiveness of antibiotics, non-antibiotic approaches, including phage therapy [14], quorum sensing inhibitors, and nanoparticle-based systems, are being explored alternatives or adjuncts to conventional treatments [15].

In conclusion, genomic characterization of *P. aeruginosa* is essential for understanding the mechanisms that enables its adaptability to the chronic pulmonary environment, and for guiding the development of alternative therapy strategies that minimize the trial-and-error approach of current interventions.
